# Promotion of human mesenchymal stem cell osteogenesis by PI3-kinase/Akt signaling, and the influence of caveolin-1/cholesterol homeostasis

**DOI:** 10.1186/s13287-015-0225-8

**Published:** 2015-12-01

**Authors:** Natasha Baker, Jihee Sohn, Rocky S. Tuan

**Affiliations:** Center for Cellular and Molecular Engineering, Department of Orthopaedic Surgery, University of Pittsburgh School of Medicine, 450 Technology Drive, Room 221, Pittsburgh, PA 15219-3143 USA; Present address: Kenneth P. Dietrich School of Arts and Sciences, Department of Biological Sciences, University of Pittsburgh, 4249 Fifth Avenue, Pittsburgh, PA 15260 USA

**Keywords:** Phosphoinositide 3-kinase/Akt, Caveolin-1, Cholesterol homeostasis, Mesenchymal stem cells, Osteogenesis, Signaling, Membrane rafts

## Abstract

**Introduction:**

Stem cells are considered an important resource for tissue repair and regeneration. Their utilization in regenerative medicine will be aided by mechanistic insight into their responsiveness to external stimuli. It is likely that, similar to all other cells, an initial determinant of stem cell responsiveness to external stimuli is the organization of signaling molecules in cell membrane rafts. The clustering of signaling molecules in these cholesterol-rich membrane microdomains can affect the activity, specificity, cross-talk and amplification of cell signaling. Membrane rafts fall into two broad categories, non-caveolar and caveolar, based on the absence or presence, respectively, of caveolin scaffolding proteins. We have recently demonstrated that caveolin-1 (Cav-1) expression increases during, and knockdown of Cav-1 expression enhances, osteogenic differentiation of human bone marrow derived mesenchymal stem cells (MSCs). The increase in Cav-1 expression observed during osteogenesis is likely a negative feedback mechanism. We hypothesize that focal adhesion signaling pathways such as PI3K/Akt signaling may be negatively regulated by Cav-1 during human MSC osteogenesis.

**Methods:**

Human bone marrow MSCs were isolated from femoral heads obtained after total hip arthroplasty. MSCs were incubated in standard growth medium alone or induced to osteogenically differentiate by the addition of supplements (β-glycerophosphate, ascorbic acid, dexamethasone, and 1,25-dihydroxyvitamin D_3_). The activation of and requirement for PI3K/Akt signaling in MSC osteogenesis were assessed by immunoblotting for phosphorylated Akt, and treatment with the PI3K inhibitor LY294002 and Akt siRNA, respectively. The influences of Cav-1 and cholesterol membrane rafts on PI3K/Akt signaling were investigated by treatment with Cav-1 siRNA, methyl-β-cyclodextrin, or cholesterol oxidase, followed by cellular sub-fractionation and/or immunoblotting for phosphorylated Akt.

**Results:**

LY294002 and Akt siRNA inhibited MSC osteogenesis. Methyl-β-cyclodextrin, which disrupts all membrane rafts, inhibited osteogenesis. Conversely, Cav-1 siRNA and cholesterol oxidase, which displaces Cav-1 from caveolae, enhanced Akt signaling induced by osteogenic supplements. In control cells, phosphorylated Akt began to accumulate in caveolae after 10 days of osteogenic differentiation.

**Conclusions:**

PI3K/Akt signaling is a key pathway required for human MSC osteogenesis, and it is likely that localization of active Akt in non-caveolar and caveolar membrane rafts positively and negatively contributes to osteogenesis, respectively.

## Introduction

New bone formation may be desirable in a variety of clinical settings, such as in osteoporosis, skeletal deformities, and in nonunion bone fractures [1]. Drugs that promote bone formation are thus in demand [2]. Alternatively, adult stem or progenitor cells, such as mesenchymal stem cells (MSCs), represent a promising resource for new bone formation via cell-based tissue engineering. These cells can be isolated from various adult tissues and have the potential to differentiate and self-renew, as well as to promote tissue repair through trophic effects on other cell types and immune-regulatory activities [3–5]. In particular, MSCs have the potential to differentiate into multiple mesenchymal lineage cells, including adipocytes, chondrocytes, and osteoblasts, underscoring the potential of MSCs for musculoskeletal regenerative medicine [6].

To utilize MSCs effectively in regenerative medicine, a clear understanding of how they respond to environmental cues is essential. Early in their genesis, MSC responses to soluble and physical environmental stimuli are likely determined, at least in part, by the availability and organization of signaling molecules at the cell surface membrane. Key cell membrane substructures involved in such signaling molecule organization are lipid microdomains known as membrane rafts. Membrane rafts are cholesterol and sphingolipid-rich liquid-ordered phases in the cell membrane that allow compartmentalization and clustering of cell surface signaling molecules [7, 8]. Such clustering promotes amplification, cross-talk, specificity, and inhibitory regulation of cell signaling. A plethora of cell signaling events are regulated in caveolae, a membrane raft subtype [9, 10]. Signal regulation in caveolae is largely attributed to the activity of their distinctive caveolin scaffolding proteins, which are essential for caveolae formation, as well as cholesterol binding and cholesterol trafficking [11–16].

There are three types of caveolin proteins, which form a hairpin loop in the cell membrane with their N-termini and C-termini remaining in the cell cytoplasm [17, 18]. Caveolin-1 (Cav-1) is most highly expressed in fat and lung tissue [19–23], but it is also expressed in many other tissues and differentiated cell types [21, 22, 24–31]. Caveolin-2 is usually coexpressed with Cav-1 and appears unable to form caveolae alone [20, 21, 32]. Caveolin-3 is highly expressed in muscle cells [22, 25, 33]. The cytoplasmic portion of Cav-1 contains a caveolin scaffolding domain (CSD) that can bind to cell signaling molecules and affect cell signal transduction, mostly in an inhibitory fashion by direct interaction or as a result of caveolae endocytosis (see reviews by [9, 34]).

We have previously shown Cav-1 expression in adult human bone marrow-derived MSCs, and that its level increases during and negatively regulates the osteogenic differentiation of MSCs [35]. This finding is consistent with the phenotype of Cav-1 null mice, which show an increased postnatal bone volume and bone formation rate [36]. Furthermore, bone marrow MSCs isolated from these mice have a greater tendency to mineralize in response to osteogenic supplements in vitro [36]. In agreement with other researchers, we hypothesize that Cav-1 expression may increase during MSC osteogenesis as a negative feedback mechanism to stabilize the phenotype of osteogenically differentiated MSCs. However, in our previous study of Cav-1 knockdown MSCs, broad gene expression analysis did not reveal any cell signaling pathway early response genes affected by Cav-1 knockdown after 24 hours of stimulation with osteogenic supplements [35]. We therefore could not discern the signaling pathways affected by Cav-1 MSCs induced to osteogenically differentiate.

Cav-1 probably dampens pro-osteogenic signaling by sequestering signaling mediators in cell surface caveolae and/or driving their internalization via caveolae endocytosis. In accordance with this idea, caveolae endocytosis of active adhesion receptors (β1 integrins) and co-localized bone morphogenetic protein (BMP) receptors contributes to the repression of osteogenesis in rat MSCs seeded on soft two-dimensional substrates [37]. Meanwhile, phosphoinositide 3-kinase (PI3K)/Akt signaling is a pathway commonly downstream of integrin signaling. Akt signaling is dependent on the generation of phosphatidylinositol 3,4,5-trisphosphate (PIP3) at the cell membrane by PI3K phosphorylation of phosphatidylinositol 4,5-bisphosphate (PIP2). We hypothesized that PI3K/Akt signaling may be pro-osteogenic and post-translationally regulated by Cav-1 and caveolae in human MSCs, because: PIP2 accumulates at the edges of caveolae [38]; Cav-1 regulates caveolae, integrin signaling, and osteogenesis; and PI3K/Akt signaling is vital for endochondral ossification [39, 40]. Here, we show that PI3K/Akt signaling is influenced by Cav-1/cholesterol homeostasis, and plays an important role in human MSC osteogenesis.

## Methods

### Tissue collection and harvesting of MSCs

Human bone marrow-derived MSCs were isolated with patient consent and Institutional Review Board approval (University of Washington, Seattle, WA, USA) from the femoral heads of patients undergoing total hip arthroplasty using a standard plastic adhesion protocol. Cells flushed from the bone marrow were pelleted and resuspended in isolation medium (α-minimal essential medium + 1× antibiotic–antimycotic, + 10 % MSC qualified fetal bovine serum (FBS) (all GIBCO/Thermo Fisher Scientific, Waltham, MA, USA) + 1 ng/ml fibroblast growth factor 2 (FGF-2; R&D Systems, Minneapolis, MN, USA), seeded into T150 flasks (Corning Inc., Corning, NY, USA), and incubated for 3–4 days at 37 °C in 5 % CO_2_, then washed twice with phosphate-buffered saline (PBS, pH 7.4) and incubated in fresh isolation medium. When colonies reached 80 % confluence, cells were reseeded at 1 × 10^6^ cells/T150 flask in isolation medium. Medium was changed every 3–4 days until cells reached 80–90 % confluency, at which time cells were either frozen or passaged. MSC isolates were routinely characterized with respect to multilineage differentiation capabilities as described previously [41].

### Cell culture and treatments

For experiments, MSCs were expanded in growth medium (high-glucose Dulbecco’s modified Eagle’s medium (DMEM) containing l-glutamine and sodium pyruvate + 1× penicillin–streptomycin + 10 % MSC qualified FBS (all GIBCO/Thermo Fisher Scientific) at 37 °C in 5 % CO_2_. Growth medium was changed every 3–4 days until cells reached 70–80 % confluence, at which time cells were seeded for experiments. For osteogenic differentiation, MSCs were seeded at 20,000 cells/cm^2^ and incubated in osteogenic medium (growth medium + 5 mM β-glycerophosphate, 50 μg/ml ascorbic acid, 10 nM dexamethasone, 10 nM 1,25-dihydroxyvitamin D3; all Sigma-Aldrich, St. Louis, MO, USA) or growth medium as a control. Medium was changed every 3 days.

For small interfering RNA (siRNA) transfection, MSCs were seeded at 20,000 cells/cm^2^, incubated overnight, washed in serum-free and antibiotic-free high-glucose DMEM, and transfected with either 50 nM Human CAV1 ON-TARGETplus SMARTpool siRNA, 100 nM Human AKT1 ON-TARGETplus SMARTpool siRNA, or 50 or 100 nM nontargeting control siRNA ON-TARGETplus Non-targeting siRNA #2 (all Dharmacon/GE Life Sciences, Lafayette, CO, USA) using DharmaFECT1 Transfection Reagent (Dharmacon/GE Life Sciences). DharmaFECT1 and each siRNA were prepared at 10× the final concentration used for transfection in serum-free and antibiotic-free high glucose DMEM and incubated for 5 minutes at room temperature. Each siRNA was then mixed 1:1 with the DharmaFECT1 and incubated for 20 minutes at room temperature. Each siRNA/DharmaFECT mix was then diluted 1:5 in antibiotic-free growth medium and added to washed MSCs at 0.13 ml/cm^2^. The final volume of DharmaFECT used was 0.08 μl/cm^2^. Twenty-four hours later, the medium was replaced with either fresh growth medium or osteogenic medium.

Disruption to all cholesterol membrane rafts was achieved by 1-hour preincubation with methyl-β-cyclodextrin (MβCD) in dimethyl sulfoxide (DMSO), used at a final concentration of 1–10 mM, before addition of control or osteogenic medium. MβCD pretreatment was performed each time the culture medium was changed during an experiment. As prolonged incubation with MβCD is cytotoxic to MSCs, MβCD was not left on cells beyond the 1-hour period, and cell viability was assessed in parallel wells using a commercial Live/Dead Assay (Life Technologies, Carlsbad, CA, USA) according to the manufacturer’s instructions. PI3K/Akt signaling was inhibited using LY294002 (Cell Signaling Technology, Danvers, MA, USA) at a final concentration of 0.5–25 μM, which remained in the culture medium for the duration of experiments, and was added fresh with each medium change. Cholesterol oxidation was achieved by 1-hour pretreatment with 0.5 U/ml cholesterol oxidase (CHOD, from *Rhodococcus* sp.; Sigma-Aldrich) before addition of osteogenic medium.

### Sucrose gradient subcellular fractionation of membrane rafts

A previously published method was used for subcellular fractionation [42]. MSCs (1,140,000 cells at seeding) were homogenized in 2 ml ice-cold 500 mM sodium carbonate pH 11.0 plus 1:100 protease inhibitor cocktail, phosphatase inhibitor cocktail 2, and phosphatase inhibitor cocktail 3 (all Sigma-Aldrich), sonicated, mixed 1:1 with 90 % sucrose in MBS buffer (25 mM 2-(N-morpholino)ethanesulfonic acid , pH 6.5, 0.15 M NaCl), and placed at the bottom of a 14 × 89 mm ultracentrifuge tube (Beckman Coulter, Brea, CA, USA). 4 ml of 35 % sucrose in 1:1 MBS:500 mM sodium carbonate was carefully layered over the homogenate, followed by 4 ml of 5 % sucrose in 1:1 MBS:500 mM sodium carbonate, and samples were centrifuged at 192,072 × *g* in a Beckman XL-70 Ultracentrifuge (SW40Ti rotor) for 22 hours. The contents of the centrifuge tubes were collected carefully in sequential 1 ml fractions from top to bottom.

### Sucrose gradient subcellular fractionation of noncaveolar and caveolar membrane rafts

Detergent-free cell lysates were prepared as already described, and were placed at the bottom of a 14 × 89 mm^2^ ultracentrifuge tube (Beckman Coulter). According to the protocol described by Yao et al. [43], 3 ml of 35 % sucrose in 1:1 MBS:500 mM sodium carbonate were layered on the lysate mixture, followed by 4 ml of 21 % sucrose in 1:1 MBS:500 mM sodium carbonate, and 1 ml of 5 % sucrose in 1:1 MBS:500 mM sodium carbonate. Samples were then centrifuged at 192,072 × *g* in a Beckman XL-70 Ultracentrifuge (SW40Ti rotor) for 22 hours. The contents of the centrifuge tubes were collected carefully in sequential 0.5 ml fractions from top to bottom, to make a total of 24 fractions.

### Immunoblotting

Cells lysates were prepared in RIPA buffer plus 1:100 protease inhibitor cocktail, phosphatase inhibitor cocktail 2, and phosphatase inhibitor cocktail 3 (all Sigma-Aldrich), and the protein concentration was determined by BCA assay (Thermo Scientific™ Pierce™ Protein Biology, Rockford, IL, USA). Equal volumes of protein (1 or 5 μg) were electrophoresed by SDS-PAGE in a 12 % polyacrylamide gel and transferred to polyvinylidene fluoride (PVDF) blots which were blocked in 5 % milk or 5 % bovine serum albumin (BSA; Sigma-Aldrich) in Tris-buffered saline + 0.05 % Tween 20 (TBST), and then incubated overnight at 4 °C with either rabbit anti-Akt or anti-serine 473 phosphorylated Akt (p-Akt) (1:1000 in TBST + 5 % BSA; both Cell Signaling Technology), or rabbit anti-Cav-1 (1:5000 in TBST + 2.5 % milk; BD Biosciences, Franklin Lakes, NJ, USA), or rabbit anti-GM130 (1:1000 in TBST + 5 % BSA; Cell Signaling Technology). Secondary antibodies were Amersham ECL donkey anti-rabbit or sheep anti-mouse horseradish peroxidase (HRP)-linked IgG antibody (both GE Healthcare UK Limited, Little Chalfont, UK). HRP activity was detected using Supersignal West Dura, Extended Duration Substrate (Thermo Scientific™ Pierce™ Protein Biology) and the chemiluminescence reaction was visualized using a FOTO/Analyst® Fx CCD imaging system (Fotodyne Inc., Hartland, WI, USA).

### Densitometric analysis of immunoblots

The intensity of bands on western blots was measured using grayscale images in Image J software (http://imagej.nih.gov/ij/). A tall, narrow, rectangle of constant shape was used to identify lanes in each western using the “Rectangular Selections” tool. The intensity of bands in each lane was then measured using the Analyze > Gels > Plot Lanes function. Peaks corresponding to bands of interest where defined from background noise using the “Straight Line” selection tool. The “Wand” tool was then used to select peaks to measure, and the Analyze > Gels > Label Peaks function used to determine peak size, which represents the intensity of the corresponding band. Band intensity values were exported to Excel 2010 (Microsoft Corporation, Redmond, WA, USA) and intensities of phosphorylated bands (e.g., p-Akt bands) normalized to the intensity of total protein signal (e.g., Pan Akt) from the same sample.

### Alkaline phosphatase assay

Cultures in 24-well plates were washed in PBS, lysed in 150 μl of 0.5 % Triton X-100 in water for 10 minutes at room temperature, and frozen and thawed twice. Then 100 μl lysate was assayed spectrophotometrically using an equal volume of substrate buffer (4 mg/ml 4-nitrophenyl phosphate (pNPP; Sigma-Aldrich)), mixed 1:1 with AMP buffer (4.8 % 2-amino-2-methyl-1-propanol (2-AMP; Sigma-Aldrich)), 2 mM MgCl_2_, pH 10.15). A_415_ values were used to calculate alkaline phosphatase (ALP) activity and expressed as a function of DNA.

### Picogreen assay for DNA

DNA was quantified using Quant-iT™ PicoGreen® dsDNA reagent (Invitrogen/Thermo Fisher Scientific, Carlsbad, CA), a fluorescent dsDNA stain (excitation 480 nm, emission 520 nm), according to the manufacturer’s instructions. A lambda phage DNA standard was used to quantify DNA from the fluorescence readings.

### Alizarin red staining

Cultures were washed in PBS, fixed in 60 % isopropanol (diluted in tap water), washed in tap water, and stained with 2 % Alizarin red S solution, pH 4.2, for calcium deposits (Rowley Biochemical Institute, Danvers, MA, USA). Cultures were washed thoroughly with tap water before scanning and imaging. Retained stain was solubilized using 0.5 ml of 10 % cetylpyridinium chloride in 10 mM Na_2_HPO_4_, pH 7.0, at room temperature for 15 minutes and quantified spectrophotometrically at 570 nm and normalized to the DNA content measured in cell lysates of duplicate wells of each sample.

### Statistical analysis

All statistical analysis was performed using Student’s two-tailed *t* test on two groups containing at least three samples each, with statistical significance at *p* ≤0.05.

## Results

**Fig. 1:**
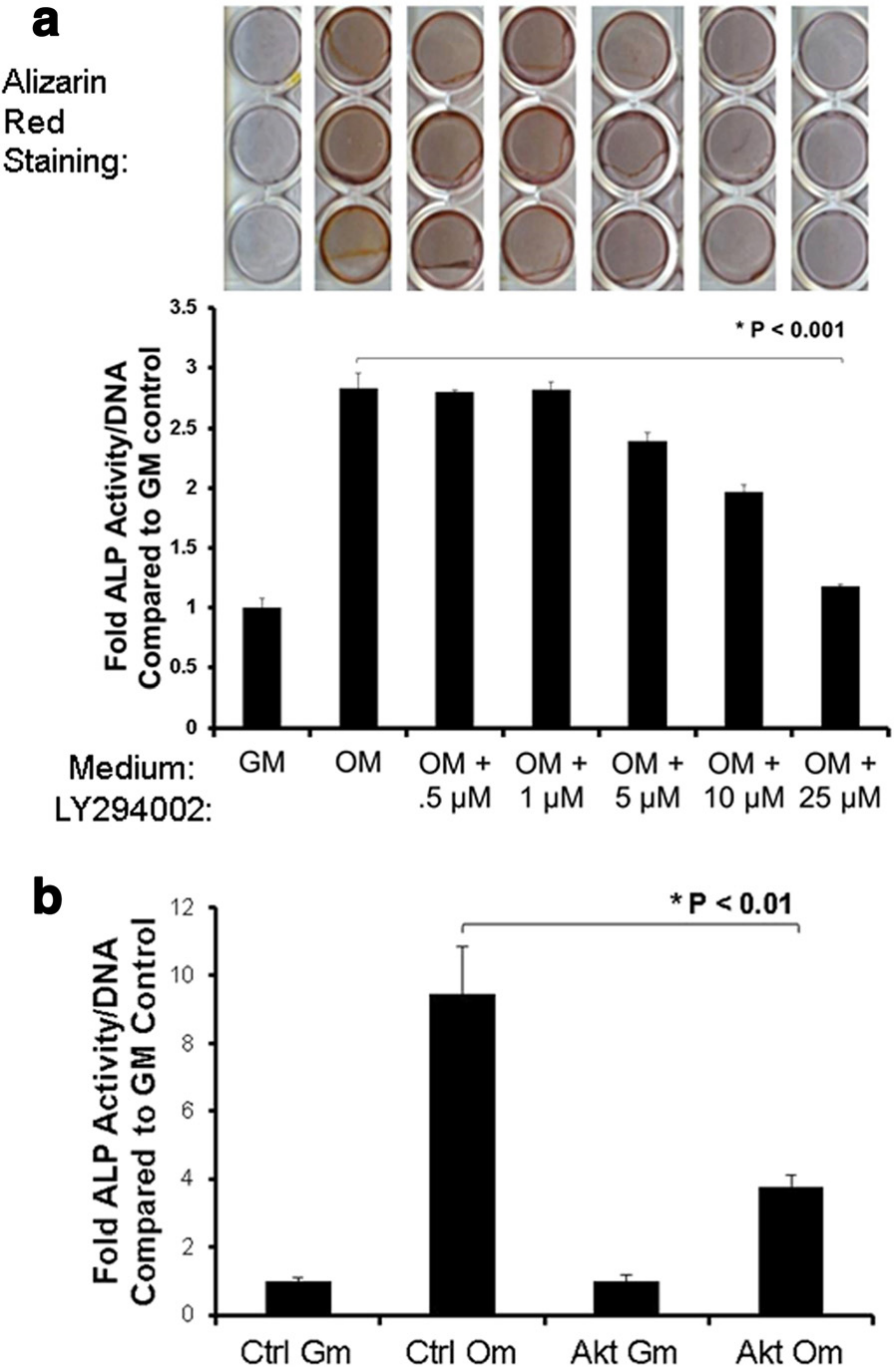
PI3K/Akt signaling is required for MSC osteogenesis. MSCs were cultured in control growth medium (*GM*) or osteogenic medium (*OM*) to induce osteogenesis. **a** Effect of LY294002. MSCs in OM were incubated in the presence or absence of different concentrations of the PI3K inhibitor LY294002, or DMSO vehicle alone. Osteogenesis was assessed by Alizarin red staining for matrix mineralization on day 21 (*top panel*, triplicate cultures shown), and measuring alkaline phosphatase (*ALP*) activity on day 4 (*bottom panel*). Values represent mean ± standard error of the mean (SEM) (*n* = 4). LY294002 inhibited osteogenesis in a dose-dependent manner, suggesting that PI3K/Akt signaling is required for human MSC osteogenesis. **b** Effect of siRNA-mediated Akt knockdown. MSCs were transfected with scramble control siRNA (*Ctrl*) or Akt siRNA before incubation in GM or OM for 4 days and measurement of ALP activity. Akt siRNA treatment significantly reduced ALP activity induced by OM. Values represent mean ± S.E.M (*n* =4).

To test our hypothesis that PI3K/Akt signaling is pro-osteogenic and regulated by Cav-1 and caveolae in human MSCs, we confirmed that PI3K/Akt signaling is required for human MSC osteogenesis, by first examining the effect of treatment with the pharmacological inhibitor of PI3K, LY294002. LY294002 dramatically inhibited MSC osteogenesis in a dose-dependent manner, as measured by ALP activity, an early marker of osteogenic differentiation, and matrix mineralization based on Alizarin red staining, which indicates terminal osteogenic differentiation (Fig. 1a). Furthermore, treatment of MSCs with Akt-specific siRNA significantly reduced induction of osteogenesis (Fig. 1b). These data indicate that PI3K/Akt signaling is required for osteogenic differentiation of human bone marrow-derived MSCs in vitro.

**Fig. 2:**
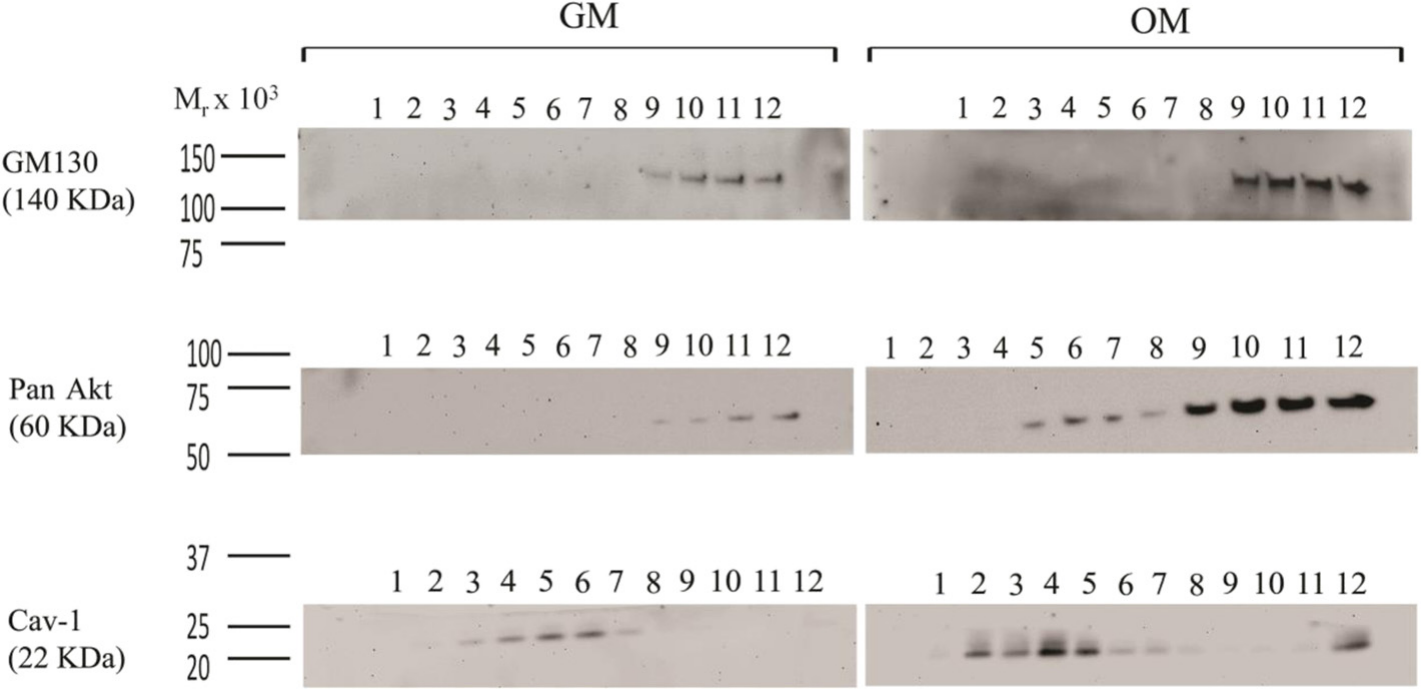
Akt is present at high levels in membrane rafts in osteogenically differentiating MSCs. A sucrose density flotation method was used to determine Akt localization to membrane rafts in MSCs cultured in either growth medium (*GM*) or osteogenic medium (*OM*). In this method, buoyant membrane rafts float to the upper fractions (4–7) of the 12-fraction sucrose gradient upon ultracentrifugation, separating from intracellular fractions that remain at the bottom of the gradient. *Top panel*: immunoblotting of the fractions for the Golgi marker GM130 indicates intracellular fractions (9–12). *Middle panel*: immunoblotting with a pan-Akt antibody shows that Akt was mostly located within intracellular fractions. However, significantly higher levels of Akt were detected in membrane rafts from cells cultured in OM on day 10. This is shown by overlapping distribution with caveolin-1 (*Cav-1*) in buoyant membrane rafts. *Bottom panel*: immunoblotting for Cav-1 indicates the presence of membrane rafts floating in the upper fractions. This result is representative of four different human bone marrow MSC donor sources tested. Molecular weight standards are shown to the left of the immunoblots

**Fig. 3:**
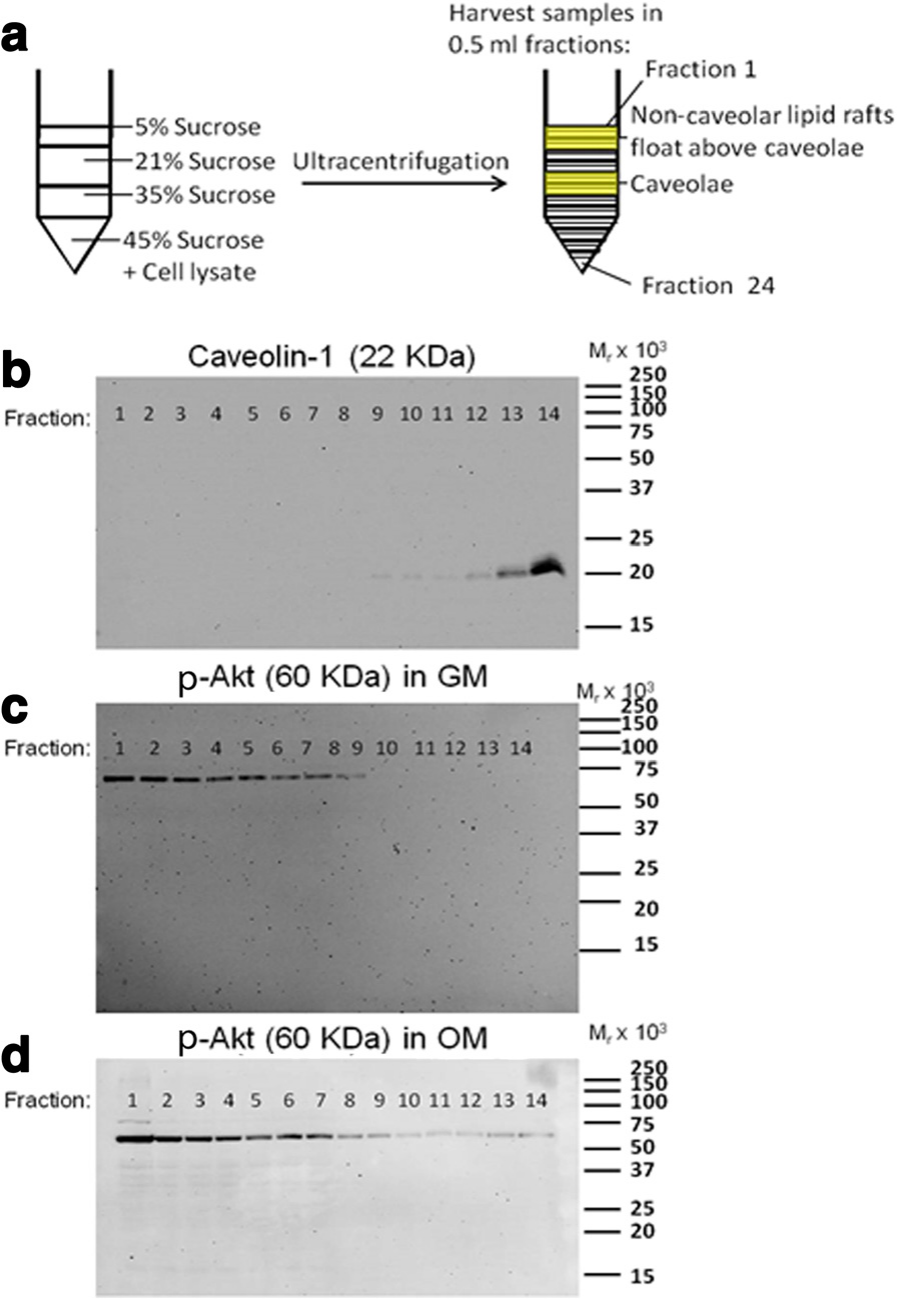
Phosphorylated Akt (*p-Akt*) is relocated to caveolae in osteogenically differentiating MSCs. **a** A modified sucrose gradient flotation method [43] was used to separate membrane rafts with different buoyancies to distinguish noncaveolar membrane rafts from caveolae. **b** Cav-1 protein was detected by immunoblotting in the middle fractions of the gradient, indicating the presence of caveolae. **c**, **d** p-Akt was much more abundant in the noncaveolar membrane raft fractions. **c** No p-Akt was detected in the caveolar fraction of MSCs cultured in standard growth medium (*GM*). **d** However, p-Akt was detected in the caveolar fraction of MSCs cultured in the presence of osteogenic supplements for 10 days (*OM*). These results suggest that a portion of p-Akt relocated to caveolae during osteogenesis. Molecular weight standards are indicated on the right of the immunoblot panels. Data shown are representative of three replicates

To begin to determine the role of membrane rafts in pro-osteogenic PI3K/Akt signaling, we probed the localization of Akt to membrane rafts during MSC osteogenesis. MSCs were cultured in control growth medium or growth medium containing osteogenesis-inducing supplements. After 10 days of culture in the presence of osteogenic supplements, MSCs express Col1a2 and higher levels of Cav-1 [35]. Lysates from MSC cultures were therefore harvested after 10 days. They were then fractionated using a previously published sucrose gradient flotation method [42]. By this fractionation method, Cav-1 protein is detected around the fifth to sixth fractions containing buoyant lipid rafts (Fig. 2) [35, 42]. Total Akt expression was probed in all gradient fractions from MSCs cultured with or without osteogenic supplements. Strikingly, a portion of Akt appeared colocalized with Cav-1 in buoyant membrane fractions in MSCs cultured with osteogenic supplements (Fig. 2). In MSCs cultured without osteogenic supplements, Akt mostly remained within intracellular fractions as indicated by the presence of the Golgi marker GM130 (Fig. 2). To investigate whether Akt accumulation in buoyant rafts was in caveolar membrane rafts specifically and whether this Akt was active, we used a modified sucrose gradient subcellular fractionation method that allows the separation of caveolar and noncaveolar rafts, and probed fractions for Ser-473 p-Akt [43]. This revealed that any buoyant fraction p-Akt in control MSCs was confined to noncaveolar membrane rafts (Fig. 3). However, incubation of MSCs in osteogenic medium caused detectable translocation of p-Akt into caveolar membrane rafts (Fig. 3).

**Fig. 4:**
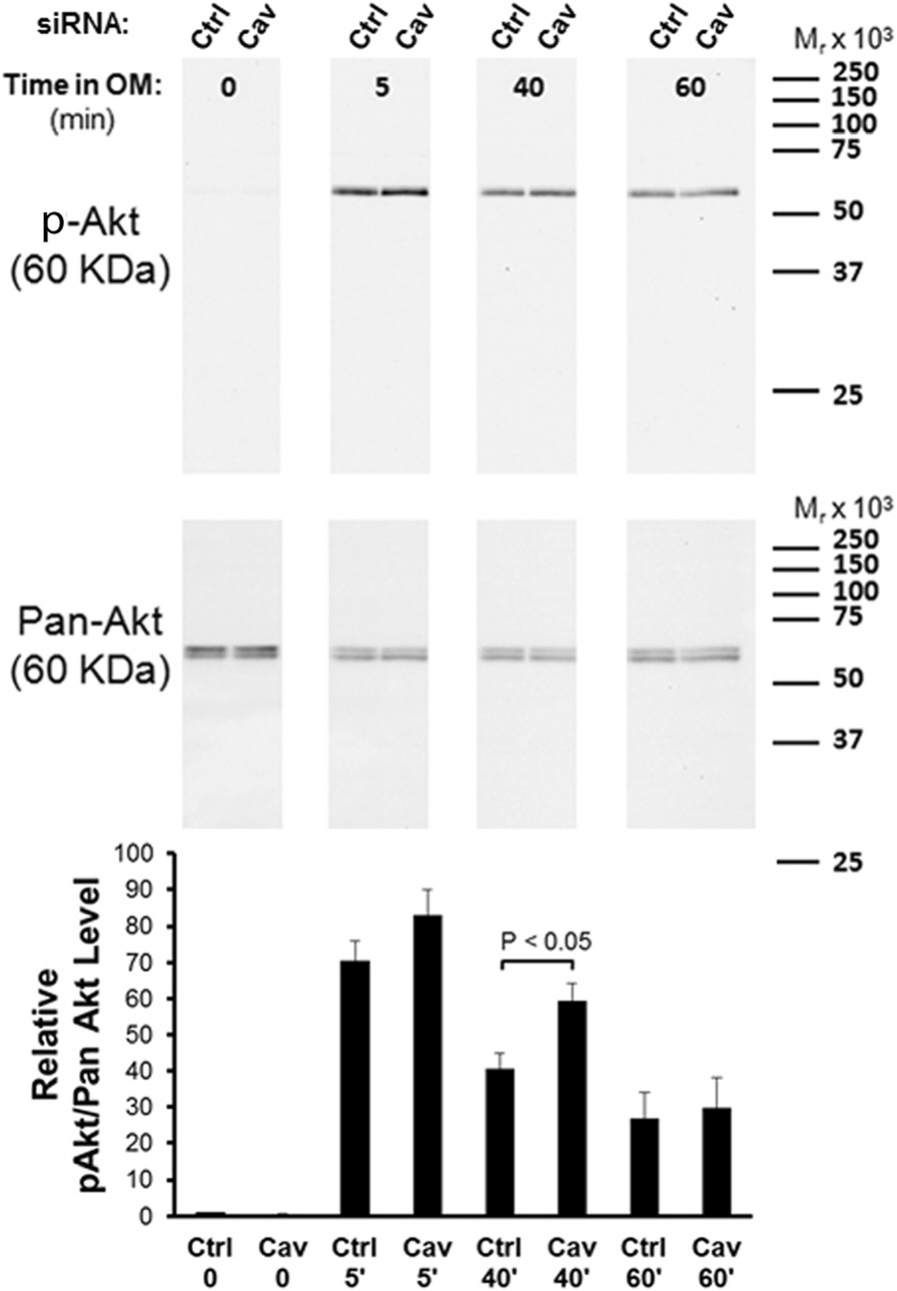
Osteogenic supplement-induced Akt phosphorylation is modestly enhanced in Cav-1 knockdown MSCs. MSCs transfected with control (*Ctrl*) or Cav-1 (*Cav*) small interfering RNA (*siRNA*) were serum starved overnight, and then stimulated with osteogenic supplements (*OM*). Immunoblot analysis of cellular proteins harvested at the indicated time points after stimulation with OM showed that Akt is phosphorylated after 5 minutes incubation in OM (*top panel*). There is a moderate increase in Akt phosphorylation in MSCs transfected with Cav-1 siRNA and incubated in OM for 5 minutes and 40 minutes compared with MSCs transfected with control nontargeting siRNA. This result is representative of three replicates with different MSC sources. The densitometric data for all replicates are presented in the bar graph (*bottom panel*), which shows the mean p-Akt/Akt signal for each sample relative to control samples in the same experiment at *t* = 0. Densitometric quantification of bands was achieved using NIH Image J software. Values represent mean ± SEM (*n* =4). The increase in p-Akt signal in MSCs transfected with Cav-1 siRNA and incubated in OM was statistically significant at 40 minutes

**Fig. 5:**
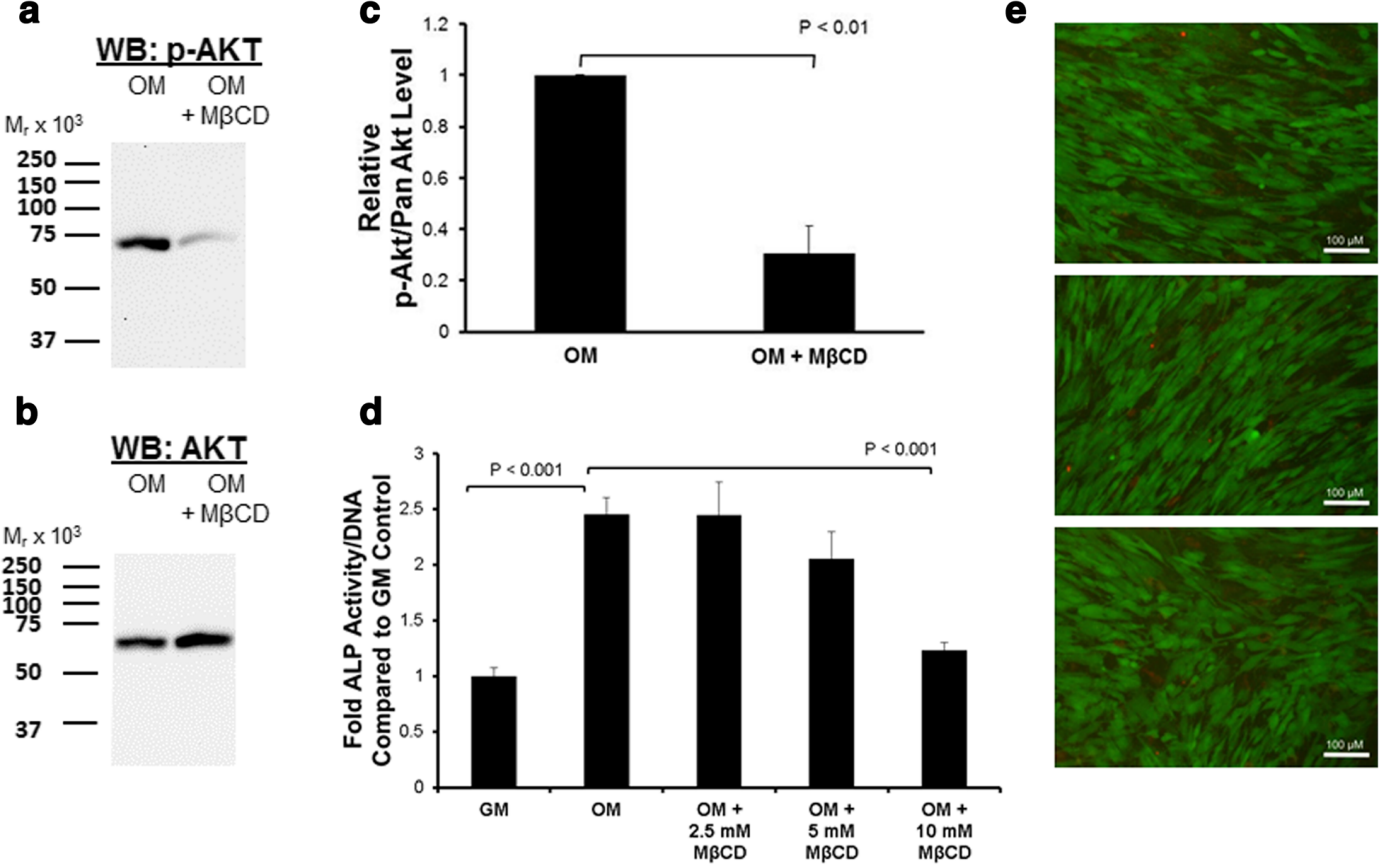
Depletion of MSC membrane cholesterol reduces Akt phosphorylation and osteogenic supplement-induced alkaline phosphatase (*ALP*) activity. MSCs were treated with standard growth medium (*GM*), treated with growth medium with osteogenic supplements (*OM*), or pretreated for 1 hour with methyl-β-cyclodextrin (*MβCD*) before addition of OM supplements. **a**, **b** Immunoblotting (western blot, WB) showed that 10 mM MβCD suppressed phosphorylation of Akt in OM (**a**), with no change in total Akt levels (**b**). Molecular weight standards are shown on the left of the immunoblot. These results are representative of three replicates with different MSC sources. **c** Densitometric analysis of the phosphorylated Akt (*p-Akt*) signal normalized to total Akt is shown for all three replicates. Values represent mean ± SEM (*n* =3). **d** Effect of MβCD treatment on OM-induced ALP activity. Significant suppression of ALP activity was seen at 10 mM MβCD. Values represent mean ± SEM (*n* = 4). **e** Three representative images from Live/Dead staining of cells exposed to 10 mM MβCD in replicate wells of samples in (**d**). Bar = 100 μm. Results shown here are representative of at least three different MSC populations studied, and suggest the requirement of membrane cholesterol for PI3K/Akt signaling and induction of osteogenesis in human MSCs

Cav-1 is known to positively interact with PI3K/Akt signaling in other, differentiated, cell types [9]. However, as Cav-1 inhibits MSC osteogenesis [35] and PI3K/Akt signaling is pro-osteogenic (Fig. 1), we postulated that the movement of p-Akt to caveolar membrane rafts during osteogenesis progression could be a negative regulatory event. To test this, we isolated protein from control siRNA and Cav-1 siRNA-transfected MSCs at multiple time points following stimulation with osteogenic supplements, and probed for Akt phosphorylation/activation by western blotting. We found that Akt was phosphorylated in MSCs stimulated with osteogenic supplements, and this activation was modestly enhanced in Cav-1 knockdown MSCs (Fig. 4). Conversely, when cells were treated with the cholesterol chelator MβCD, which strips cholesterol from both caveolar and noncaveolar membrane rafts, Akt phosphorylation was inhibited (Fig. 5). These results suggest that localization of Akt in noncaveolar lipid rafts is important for osteogenesis. Accordingly, MβCD dose-dependently inhibited ALP activity induced after 4 days of incubation of MSCs with osteogenic supplements (Fig. 5). We confirmed that these affects were not due to cytotoxicity by performing Live/Dead staining in parallel cultures (Fig. 5e). Cholesterol-rich membrane rafts are therefore required for Akt phosphorylation and MSC osteogenesis, while movement of p-Akt to caveolae lipid rafts could be inhibitory for pro-osteogenic Akt signaling.

**Fig. 6:**
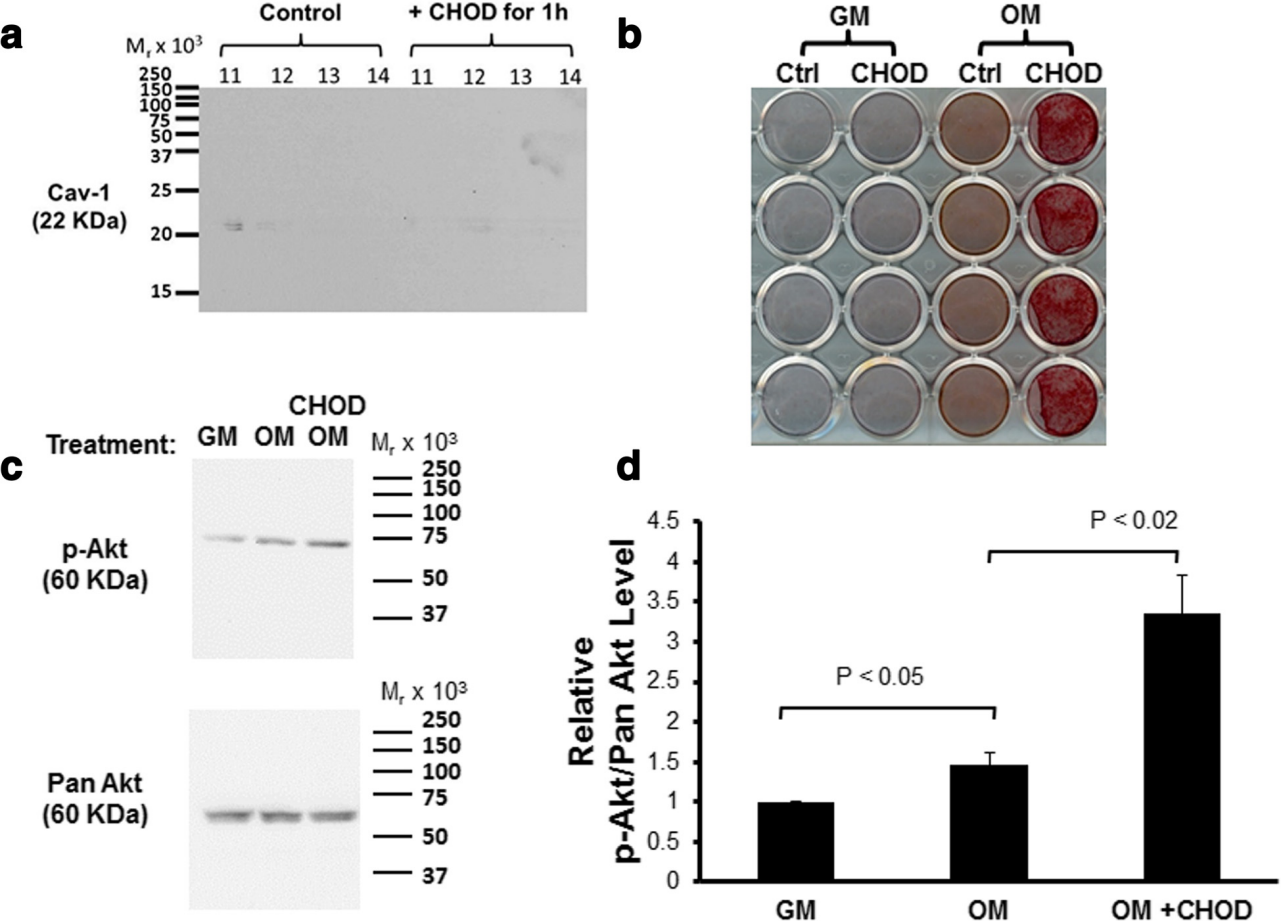
Cholesterol oxidase (*CHOD*) treatment enhances MSC osteogenesis. **a** MSCs were treated for 1 hour with or without CHOD, and then caveolae were isolated by sucrose gradient flotation. Fractions expected to contain caveolae (see Fig. 3) were immunoblotted for caveolin-1 (*Cav-1*). Results show that CHOD pretreatment displaces Cav-1 from buoyant caveolae membrane rafts. **b**, **c** MSCs were treated with standard growth medium (*GM*), treated with growth medium with osteogenic supplements (*OM*), or pretreated for 1 hour with CHOD before addition of growth medium with osteogenic supplements (CHOD). **b** After continuous treatment for 10 days with medium change every 3 days, Alizarin red staining revealed that OM-induced matrix mineralization was accelerated in CHOD-treated MSCs (quadruplicate cultures shown). **c** Immunoblotting showed that 5 minutes after initial CHOD treatment, phosphorylated Akt (*p-Akt*) levels increased, while total Akt (Pan Akt) remained unchanged. These results are representative of three replicates with different MSC sources. **d** Densitometric analysis is shown for all three replicates. Values represent mean ± SEM (*n* =3). This confirmed a significant increase in p-Akt/Akt levels upon CHOD treatment, suggesting that pretreatment with CHOD enhances OM-mediated induction of PI3K/Akt signaling in MSCs. *Ctrl* control

To further test how caveolar lipid rafts affect Akt activity, we attempted to displace Cav-1 from caveolae by means of cholesterol oxidation. Treatment of cells with CHOD has been reported to cause the reversible relocation of Cav-1 from cell surface caveolae to the Golgi apparatus in fibroblasts [44]. We confirmed that treatment with CHOD caused Cav-1 to move out of caveolae in MSCs (Fig. 6a). When this pretreatment was administered for the duration of osteogenic supplement-induced osteogenesis, matrix mineralization was enhanced (Fig. 6b). Furthermore, when MSCs were pretreated with CHOD, Akt phosphorylation in response to osteogenic supplements was enhanced (Fig. 6c, d). Taken together, our results suggest that Akt activation is important for osteogenic supplement-induced MSC osteogenesis, and that noncaveolar lipid rafts are required for this signaling, while Akt localization to Cav-1-containing caveolae lipid rafts may suppress Akt activation and osteogenic signaling.

## Discussion

**Fig. 7:**
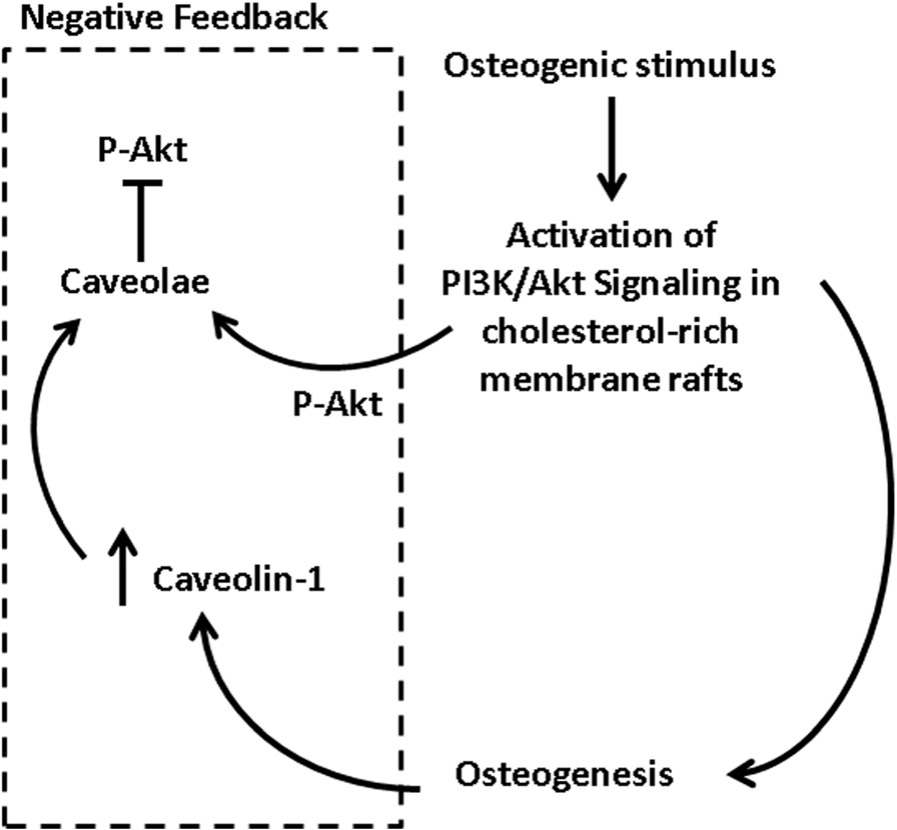
Proposed inhibitory feedback loop for MSC osteogenesis. We propose that phosphoinositide 3-kinase (*PI3K*)/Akt activation is a key signaling event required for the induction of osteogenesis in human bone marrow MSCs. As osteogenesis progresses, Cav-1 is upregulated as a negative feedback mechanism to suppress continued osteogenesis. PI3K/Akt signaling is one pathway that Cav-1 suppresses within caveolar membrane rafts to achieve this end, and as osteogenesis progresses more phosphorylated Akt (*p-Akt*) is translocated to this subcellular compartment

Previously, we reported that siRNA-mediated knockdown of Cav-1 expression in human MSCs enhances MSC osteogenesis [35]. Here, we show that Akt is phosphorylated in MSCs stimulated with osteogenic supplements and that this is enhanced in Cav-1 siRNA-transfected MSCs. We also show that PI3K/Akt signaling is essential for human MSC osteogenesis in a manner dependent on cholesterol-rich membrane rafts, and that p-Akt translocates to caveolar membrane rafts during osteogenesis. Furthermore, CHOD treatment displaces Cav-1 from caveolae, enhancing p-Akt signaling and MSC osteogenesis. Therefore, p-Akt localization to caveolae may act as an inhibitory negative feedback mechanism to suppress continued MSC osteogenesis. Taken together, our results suggest a pathway in which PI3K/Akt signaling is required for human MSC osteogenic differentiation, during the progression of which Cav-1 expression increases [35], and p-Akt relocates to Cav-1-containing caveolar membrane rafts, thus contributing to the suppression of continued osteogenesis (Fig. 7).

In mice, there is substantial evidence that PI3K/Akt signaling is important for osteogenesis and endochondral ossification (reviewed in [45]). Knockout of Akt negatively affects murine endochondral ossification [39, 40]. Similarly, longitudinal bone growth in mouse embryonic tibiae is suppressed by inhibition of PI3K/Akt signaling using the same inhibitor as used here, LY294002 [46]. Akt activity is also required for the growth of isolated mouse metatarsal bones in vitro both during the chondrogenic phase of the process and for osteoblast maturation [47].

There is also substantial evidence that PI3K/Akt signaling is required for murine osteogenesis from in vitro cell culture studies. In cultures of 2T3 osteoblast precursor cells isolated from mouse calvaria, BMP-induced osteogenesis requires PI3K/Akt signaling [48]. This is also true for osteogenesis of mouse embryonic fibroblasts (MEFs), murine bone marrow-derived MSCs, and in the mouse MSC line C3H10T1/2 [47, 49]. Studies in murine cells also suggest that PI3K/Akt is required early in the transcriptional activation of osteogenesis. For example, in an immature osteoblastic cell line derived from mouse calvaria (MC3T3-E1), Runx2 overexpression upregulates expression of the PI3K subunits p85 and p110β, as well as Akt protein in these cells, and reciprocally PI3K/Akt signaling is required for the DNA binding and transcriptional activities of Runx2 [50]. It therefore appears that Runx2 and PI3K/Akt signaling act in a positive feedback loop to induce osteogenesis, and PI3K/Akt signaling is essential for the induction of Runx2 expression and activity. The exact mechanism for this is unknown, however, because Akt does not appear to directly phosphorylate Runx2. Nevertheless, PI3K/Akt signaling is required for Runx2-induced osteogenesis of C3H10T1/2 cells [48], and BMP-2-induced expression of Runx2 in C3H10T1/2 cells and mouse bone marrow stromal cells [47, 49]. Furthermore, in 2T3 cells, Akt may be required for translocation of BMP signaling intermediates, the Smads, to the cell nucleus [48]. Here, we have shown that PI3K/Akt signaling is required for the osteogenic differentiation of human bone marrow-derived MSCs in vitro. Furthermore, we show that this signaling is influenced by cholesterol-rich membrane rafts.

Knockdown of Cav-1 expression and CHOD treatment in human MSCs enhanced Akt activation in response to osteogenic supplements. CHOD produces oxysterols by cholesterol oxidation. In turn, oxysterols are known to suppress Cav-1 expression and cause Cav-1 translocation out of caveolae [44, 51] and to promote MSC osteogenesis [52]. Our findings reported here therefore agree with a pathway in which PI3K/Akt signaling positively contributes to MSC osteogenesis, while Cav-1 expression increases during osteogenesis and, via caveolae, dampens this pathway as one means to suppress continued osteogenesis (Fig. 7).

Meanwhile, our results also suggest that some membrane cholesterol is required for Akt activation and osteogenesis. This is probably required for Akt activation in noncaveolar membrane rafts (Fig. 3). In agreement with a requirement for cholesterol in osteogenesis, others have found that some cholesterol biosynthesis inhibitors (statins) can inhibit the osteogenesis of mouse marrow stromal cells (M2-10B4 cells), and this can be rescued by supplementation with the cholesterol biosynthesis intermediate, mevalonic acid, which is produced downstream of statin inhibition [53].

It must be noted that statins have also been shown to enhance MSC osteogenesis in vitro [54, 55]. There is also evidence indicating that systemic statin treatment enhances tissue repair, particularly in bone [56]. This may still agree with our hypothetical pathway in Fig. 7, due to the dual role of cholesterol in both noncaveolae and caveolae rafts. Intracellular cholesterol levels and Cav-1/caveloae activity are directly, functionally linked; an increase in intracellular cholesterol levels promotes Cav-1-directed cholesterol efflux and caveolae formation, and internalization of free cholesterol promotes Cav-1 expression [51, 57]. Therefore, if intracellular cholesterol levels are below a certain threshold through statin treatment, cell surface caveolae levels may be low, thus preventing Cav-1/caveolae suppression of continued osteogenesis. However, in mouse osteoblasts it has been shown that statins can directly activate osteogenic PI3K/Akt signaling via membrane-associated Ras [58]. The pro-osteogenic effects of statins may therefore not be completely attributed to their cholesterol biosynthetic inhibitory activities.

## Conclusions

Similar to the scenario in mice, PI3K/Akt is an essential pathway for human MSC osteogenesis. Noncaveolar cholesterol-rich lipid rafts appear to be important for activation of PI3K/Akt signaling and osteogenesis. However, Cav-1, the scaffolding protein of caveolae membrane rafts that suppresses osteogenesis in both murine and human MSCs, may suppress PI3K/Akt signaling within caveolae.

## Abbreviations

ALP: Alkaline phosphatase
BMP: Bone morphogenetic protein
BSA: Bovine serum albumin
Cav-1: Caveolin-1
CHOD: Cholesterol oxidase
CSD: Caveolin scaffolding domain
DMEM: Dulbecco’s modified Eagle’s medium
DMSO: Dimethyl sulfoxide
FBS: Fetal bovine serum
FGF2: Fibroblast growth factor 2
HRP: Horseradish peroxidase
MSC: Mesenchymal stem cell
MβCD: Methyl-β-cyclodextrin
p-Akt: Phosphorylated Akt
MEF: Mouse embryonic fibroblast
PBS: Phosphate-buffered saline
PI3K: Phosphoinositide 3-kinase
PIP3: Phosphatidylinositol 3,4,5-trisphosphate
PIP2: Phosphatidylinositol 4,5-bisphosphate
SEM: Standard error of the mean
siRNA: Small interfering RNA
TBST: Tris-buffered saline + 0.05 % Tween 20.
